# Balanced Salt Solution Versus Ringer Lactate in Emergency Laparotomy: A Prospective Randomized Study of Acid-Base and Electrolyte Outcomes

**DOI:** 10.7759/cureus.109470

**Published:** 2026-05-22

**Authors:** Ramik Chaudhary, Nandita Chaudhary, Sumit Sachan, Yashpal Singh

**Affiliations:** 1 Department of Anaesthesiology, Banaras Hindu University, Varanasi, IND; 2 Department of Pathology, Sanjay Gandhi Post Graduate Institute of Medical Sciences, Lucknow, IND; 3 Department of Anaesthesiology, Sanjay Gandhi Post Graduate Institute of Medical Sciences, Lucknow, IND

**Keywords:** balanced salt solution, emergency laparotomies, intraoperative fluid, resuscitation, ringer lactate

## Abstract

Background

Emergency laparotomy is associated with significant physiological derangements, where optimal fluid therapy plays a crucial role in maintaining acid-base balance and electrolyte homeostasis. The choice of intraoperative crystalloid may influence perioperative outcomes.

Objectives

To compare the efficacy and safety of balanced salt solution (BSS) and Ringer lactate (RL) on acid-base status and electrolyte profile in patients undergoing emergency laparotomy.

Methods

This prospective randomized study was conducted at a tertiary care center and included 60 patients with intestinal obstruction or perforation undergoing emergency laparotomy. Patients were randomly allocated into two groups to receive either BSS or RL as intraoperative maintenance fluid. Arterial blood gas parameters and serum electrolytes were assessed perioperatively and compared between the groups.

Results

BSS was associated with a more stable acid-base profile, with higher pH and bicarbonate levels compared to RL. Serum sodium levels were mildly higher in the BSS group, while potassium levels were higher in the RL group. Chloride levels remained comparable between the groups. Serum calcium levels were significantly higher in the BSS group. Lactate levels decreased in the BSS group but increased significantly in the RL group. Serum osmolarity remained within normal limits in both groups.

Conclusion

BSS demonstrated superior maintenance of acid-base balance and electrolyte stability compared to RL in patients undergoing emergency laparotomy. Its use may contribute to improved perioperative metabolic outcomes.

## Introduction

Emergency laparotomy remains a cornerstone of surgical practice and is associated with significant morbidity and mortality, particularly in resource-limited settings. Optimal perioperative management, especially fluid therapy, plays a critical role in influencing patient outcomes. Even subtle differences in the composition of intravenous fluids can significantly affect acid-base balance, electrolyte status, and overall physiological stability, as demonstrated by Self et al. [1].

Emergency laparotomies encompass a wide spectrum of conditions, including trauma, intestinal obstruction, and perforation, with non-trauma cases being more prevalent in low- and middle-income countries. These patients often present with complex physiological derangements such as hypovolemia, metabolic acidosis, electrolyte imbalance, hypothermia, and coagulopathy. The presence of sepsis or peritonitis further complicates management, necessitating a judicious and individualized approach to fluid therapy.

Crystalloid solutions remain the mainstay of intraoperative fluid resuscitation. Ringer lactate (RL) has been widely used due to its availability and cost-effectiveness; however, concerns persist regarding lactate metabolism and its potential contribution to acid-base disturbances in critically ill patients, as reported by Jahangir et al. [2] and Huang et al. [3]. In contrast, balanced salt solutions (BSSs) more closely resemble plasma electrolyte composition and have been shown to better preserve acid-base homeostasis and reduce metabolic derangements compared to unbalanced solutions, as demonstrated by Pfortmueller et al. [4] and Brahma et al. [5].

Several studies and meta-analyses have highlighted the advantages of balanced crystalloids in maintaining acid-base equilibrium and electrolyte stability in surgical and critical care settings. Dey et al. [6] demonstrated improved metabolic profiles with balanced solutions in neurosurgical patients, while Yang et al. [7] reported better perioperative biochemical stability with balanced fluids in non-cardiac surgery. Additionally, Kannan et al. [8] emphasized the importance of optimized fluid strategies in improving perioperative outcomes in high-risk surgical populations.

Despite the growing body of evidence favoring balanced crystalloids, limited data exist specifically in the setting of emergency laparotomy, where patients often present with profound physiological derangements. Given the unique challenges in this population, the choice of intraoperative fluid may have important implications for perioperative outcomes. Previous randomized studies, such as that by Saini et al. [9] in renal transplant patients, have demonstrated improved acid-base balance with balanced crystalloids compared to conventional solutions. However, evidence in emergency surgical populations remains scarce.

Therefore, this prospective randomized study was conducted to compare the effects of BSS and RL on perioperative acid-base status and electrolyte balance in patients undergoing emergency laparotomy for intestinal obstruction and perforation. By addressing this clinically relevant question, the study aims to contribute to evidence-based fluid selection and improve perioperative care in emergency surgical settings.

## Materials and methods

The present prospective randomized study was conducted in the operation theater complex of the Department of Anaesthesiology at a tertiary care hospital between October 2021 and September 2023. The study protocol was approved by the Institutional Ethics Committee and registered with the Clinical Trials Registry of India (CTRI/2022/01/039159). Written informed consent was obtained from all participants prior to enrolment.

Study design

A prospective randomized double-blind study.

Patient selection criteria: Inclusion and exclusion criteria

Patients aged between 18 and 60 years who were received for emergency surgery within 24 hours of admission following initial resuscitation were included in the study. Patients were excluded if there was a refusal by the patient or their relatives to participate. Additionally, patients receiving high inotropic support or ongoing blood transfusion were excluded. Those with pre-existing systemic illnesses such as diabetes mellitus, hypertension, chronic respiratory disease, anemia, or chronic kidney disease were also not considered. Patients with end-stage organ damage, including hepatic, renal, or cardiovascular disorders, were excluded from the study. Furthermore, individuals presenting with severe metabolic derangements, defined as pH <7.20 or serum sodium <125 mEq/L, and patients with abdominal trauma were excluded.

Patients were randomized into two equal groups (n=30 each) using computer-generated random numbers and sealed opaque envelopes. The participants and investigators involved in data collection and outcome assessment were blinded to group allocation, which was revealed only after completion of data analysis.

Methodology

This prospective randomized study included patients undergoing emergency exploratory laparotomy for intestinal obstruction or perforation. Following initial resuscitation according to institutional protocols, baseline investigations, including arterial blood gas (ABG) analysis, were performed. After obtaining written informed consent, 60 eligible patients were enrolled and randomized into two groups of 30 patients each. Demographic and baseline clinical parameters were recorded for all participants. All patients underwent surgery under general anesthesia with epidural catheter placement for postoperative analgesia. Based on randomization, patients were assigned to one of the following groups:

Group R: Received RL as the intraoperative maintenance fluid and continued up to 6 hours postoperatively.

Group B: Received BSS as the intraoperative maintenance fluid and continued up to 6 hours postoperatively.

Intraoperative fluid therapy was administered according to calculated fluid requirements, considering preoperative deficits, blood loss, third-space losses, and urine output. ABG analysis was performed preoperatively, immediately postoperatively (after extubation), and 6 hours postoperatively. Parameters assessed included pH, base excess, serum bicarbonate, sodium, potassium, chloride, lactate, and serum osmolality, which were compared between the two groups. Urine output was monitored throughout the perioperative period. Depending on their perioperative clinical condition, patients were either extubated at the conclusion of surgery, transferred to the postoperative ward with ventilatory support, or shifted to the intensive care unit for further postoperative management.

Data collection

**Figure 1:**
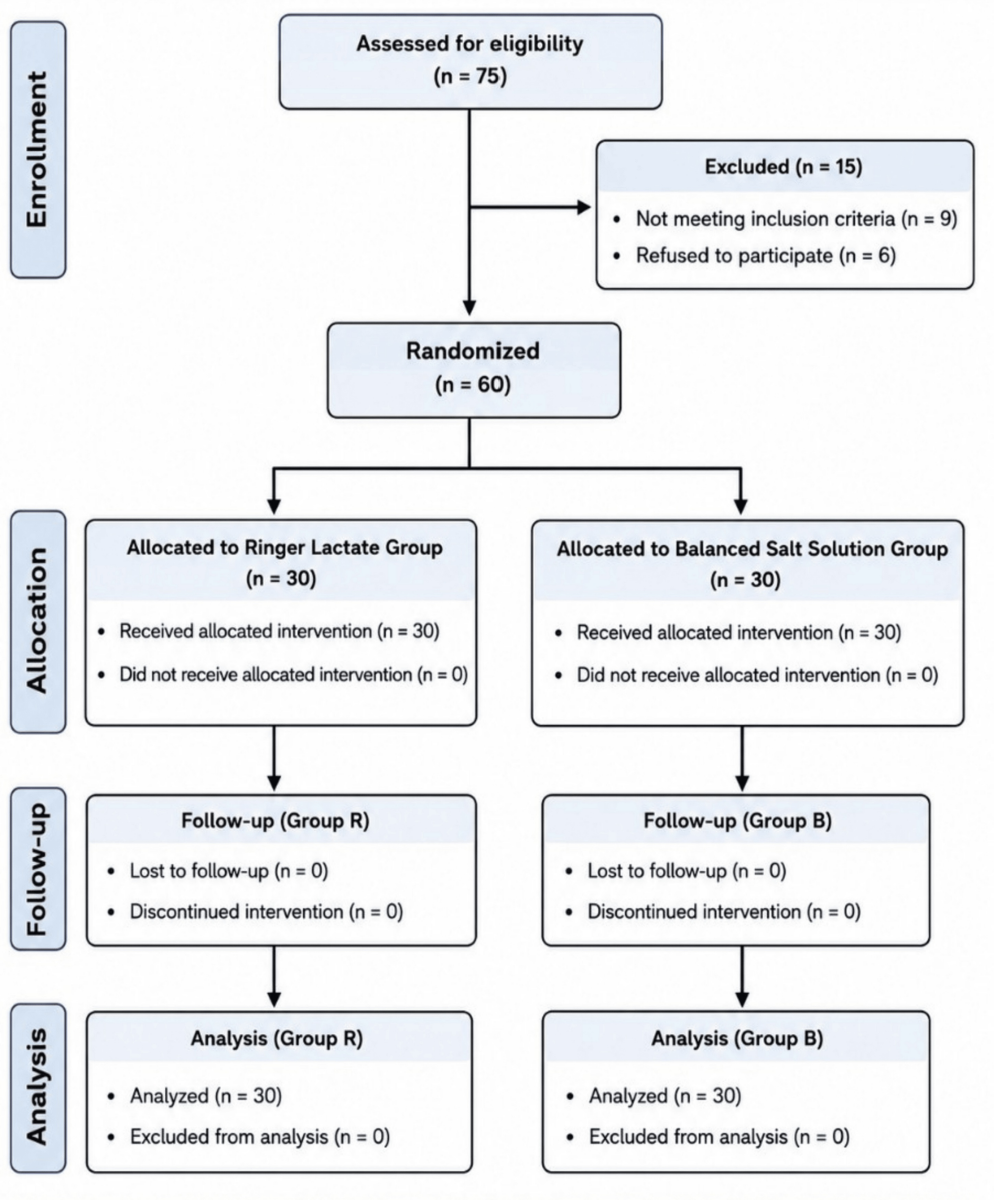
Consort flow diagram of patient's enrollment, allocation, follow-up, and analysis

Data were collected from routine patient care records in the SSH emergency operating theater. This included information on demographic characteristics, primary diagnosis, and associated comorbidities (if any), and Glasgow Coma Scale (GCS) scores were recorded, as originally described by Teasdale and Jennett [10]. Perioperatively, details regarding the type and volume of intravenous fluids administered, acid-base parameters from ABG analysis, and urine output were recorded. All data were systematically charted and evaluated during the perioperative period. The final clinical outcome for each patient was subsequently documented (Figure 1).

Outcome measure

The primary outcome of the study was a comparison of acid-base and electrolyte status between two groups.

Sample size calculation

The sample size was calculated using the software Power Analysis and Sample Size Version 16 (PASS-16, NCSS, LLC, Kaysville, UT, USA). As no previously published study with an identical methodology and primary outcome was available at the time of protocol development, the effect size and standard deviation were derived from preliminary pilot observations conducted at our institute. Based on these pilot data, a 20% difference in the primary outcome between the two groups, with an estimated standard deviation of 25%, was considered clinically significant. Using a power of 80% and a two-sided alpha error of 5%, the minimum required sample size was calculated to be 26 patients per group. To account for possible dropouts and incomplete data, 30 patients were enrolled in each group.

Statistical evaluation

Data were expressed as numbers, percentages, and mean ± standard deviation. The distribution of variables was assessed for normality using the Kolmogorov-Smirnov test along with Q-Q plot analysis. Continuous variables were compared using the unpaired two-tailed Student’s t-test, while categorical variables were analyzed using Pearson’s chi-square test. A p-value <0.05 was considered statistically significant.

## Results

A total of 60 patients were enrolled and randomized into two groups (n=30 each). The two groups were comparable with respect to baseline characteristics. The mean age in Group R and Group B was 41.5 ± 14.19 years and 35.83 ± 10.56 years, respectively, with no statistically significant difference (p = 0.084). Gender distribution, body weight, and GCS scores were also comparable between the groups (p > 0.05) (Table 1).

**Table 1:** Baseline characteristics of patients in the two groups Data are expressed as mean ± SD for continuous variables and n (%) for categorical variables. Continuous variables were compared using the unpaired Student’s t-test, and categorical variables were analyzed using the chi-square test. Test statistics: Age (t = X, p = 0.084), Weight (t = X, p = 0.16), Gender (χ² = X, p = 0.29), GCS score (χ² = X, p = 0.15). A p-value <0.05 was considered statistically significant.

Parameter	Group R (n = 30)	Group B (n = 30)	p-Value
Age (years) (mean ± SD)	41.5 ± 14.19	35.83 ± 10.56	0.084
Age distribution (n (%))			
18-35 years	11 (36.67%)	16 (53.33%)	
36-50 years	10 (33.33%)	11 (36.67%)	
>50 years	9 (30.00%)	3 (10.00%)	
Gender (n (%))			0.29
Male	16 (53.33%)	20 (66.67%)	
Female	14 (46.67%)	10 (33.33%)	
Weight (kg) (mean ± SD)	56.96 ± 8.72	59.93 ± 7.43	0.16
GCS score (n (%))			0.15
14	2 (6.67%)	0 (0%)	
15	28 (93.33%)	30 (100%)	

Baseline ABG parameters and serum electrolytes were comparable between the two groups (p > 0.05), indicating homogeneity at study entry (Table 2).

**Table 2:** Comparison of serum electrolytes between the two groups at different time intervals Data are expressed as mean ± SD. Intergroup comparisons were performed using the unpaired Student’s t-test. Corresponding t-scores and p-values were calculated for all parameters. Significant differences were observed for sodium (6 hours: t = 2.08, p = 0.042), potassium (6 hours: t = 3.34, p = 0.0015), and calcium (6 hours: t = 2.62, p = 0.012). A p-value <0.05 was considered statistically significant.

Parameter	Time Point	Group R (n = 30)	Group B (n = 30)	p-Value
Sodium (mEq/L) (mean ± SD)	Preoperative	137.16 ± 6.04	137.76 ± 5.96	0.70
	Postoperative	137.00 ± 4.14	138.00 ± 3.51	0.31
	6 hours postoperative	136.86 ± 4.19	138.73 ± 2.59	0.042
Potassium (mEq/L) (mean ± SD)	Preoperative	3.59 ± 0.59	3.67 ± 0.62	0.61
	Postoperative	3.57 ± 0.42	3.73 ± 0.40	0.15
	6 hours postoperative	3.50 ± 0.40	3.80 ± 0.29	0.0015
Chloride (mEq/L) (mean ± SD)	Preoperative	105.30 ± 5.08	104.83 ± 5.38	0.72
	Postoperative	105.75 ± 3.45	104.87 ± 4.16	0.37
	6 hours postoperative	105.87 ± 3.44	105.00 ± 3.89	0.36
Calcium (mmol/L) (mean ± SD)	Preoperative	1.05 ± 0.089	1.05 ± 0.083	1.00
	Postoperative	1.07 ± 0.066	1.08 ± 0.068	0.56
	6 hours postoperative	1.07 ± 0.05	1.10 ± 0.04	0.012

Acid-base parameters

The pH values were comparable preoperatively and immediately postoperatively between the groups (p > 0.05). However, at 6 hours postoperatively, Group B demonstrated a significantly higher pH compared to Group R (7.38 ± 0.02 vs 7.35 ± 0.03; p < 0.0001), suggesting better acid-base status with BSS (Table 3).

**Table 3:** Comparison of pH of patients between the two groups at different time intervals Data are expressed as mean ± SD. Intergroup comparisons were performed using the unpaired Student’s t-test. Corresponding t-scores and p-values for pH were preoperative (t = 1.09, p = 0.28), postoperative (t = 1.11, p = 0.271), and 6 hours postoperative (t = 4.56, p < 0.0001). A p-value <0.05 was considered statistically significant.

pH	Group R (n = 30)	Group B (n = 30)	p-Value
	Mean ± SD	Mean ± SD	
Preoperative	7.35 ± 0.07	7.34 ± 0.07	0.28
Postoperative	7.36 ± 0.04	7.37 ± 0.03	0.271
6 h postoperative	7.35 ± 0.03	7.38 ± 0.02	<0.000

Base excess remained comparable between the groups at all measured time points (p > 0.05) (Table 4).

**Table 4:** Comparison of base excess of patients between the two groups at different time intervals Data are expressed as mean ± SD. Intergroup comparisons were performed using the unpaired Student’s t-test. Corresponding t-scores and p-values for base excess were preoperative (t = 1.02, p = 0.31), postoperative (t = 0.25, p = 0.80), and 6 hours postoperative (t = 0.78, p = 0.44). A p-value <0.05 was considered statistically significant.

Base Excess	Group R (n=30)	Group B (n=30)	p-Value
	Mean ± SD	Mean ± SD	
Preoperative	1.02 ± 4.66	-0.3 ± 5.47	0.31
Postoperative	2.21 ± 2.76	2.06 ± 1.87	0.8
6 h postoperative	2.11 ± 2.49	1.7 ± 1.55	0.44

Electrolyte profile

Serum sodium levels were comparable preoperatively and immediately postoperatively (p > 0.05) but were significantly higher in Group B at 6 hours postoperatively (138.73 ± 2.59 vs 136.86 ± 4.19; p = 0.042). Serum potassium levels were significantly higher in Group B at 6 hours postoperatively (3.80 ± 0.29 vs 3.50 ± 0.40; p = 0.0015), while remaining comparable at earlier time points. Serum chloride levels did not differ significantly between the groups at any time point (p > 0.05). Serum calcium levels were comparable preoperatively and immediately postoperatively, but showed a statistically significant increase in Group B at 6 hours postoperatively (p = 0.012) (Table 2).

Serum bicarbonate (HCO₃⁻) levels were significantly higher in Group B both immediately postoperatively (23.16 ± 1.13 vs 21.55 ± 1.2; p < 0.0001) and at 6 hours postoperatively (23.84 ± 0.98 vs 22.25 ± 1.11; p < 0.0001), indicating improved metabolic profile in patients receiving BSS (Table 5).

**Table 5:** Comparison of HCO3/serum bicarbonate of patients between the two groups at different time intervals Values are presented as mean ± SD. Comparisons between the two groups were analyzed using the unpaired Student’s t-test. The calculated t-scores and corresponding p-values for HCO₃⁻ were preoperative (t = 0.35, p = 0.73), postoperative (t = 5.37, p < 0.0001), and 6 hours postoperative (t = 5.87, p < 0.0001). Statistical significance was considered at p <0.05.

HCO_3_	Group R (n = 30)	Group B (n = 30)	p-Value
	Mean ± SD	Mean ± SD	
Preoperative	20.88 ± 1.9	20.71 ± 2	0.73
Postoperative	21.55 ± 1.2	23.16 ± 1.13	<0.0001
6 h postoperative	22.25 ± 1.11	23.84 ± 0.98	<0.0001

Lactate and osmolarity

Serum lactate levels were comparable preoperatively (p = 0.080) but were significantly higher in Group R both immediately postoperatively (3.23 ± 0.87 vs 2.54 ± 0.58; p = 0.0006) and at 6 hours postoperatively (2.64 ± 0.78 vs 2.15 ± 0.55; p = 0.0067) (Table 6). 

**Table 6:** Comparison of serum lactate of patients in both groups Data are expressed as mean ± SD. Intergroup comparisons for serum lactate levels were performed using the unpaired Student’s t-test. The corresponding t-scores and p-values were preoperative (t = 1.79, p = 0.0803), postoperative (t = 3.62, p = 0.0006), and 6 hours postoperative (t = 2.82, p = 0.0067). A p-value <0.05 was considered statistically significant.

Lactate	Group R (n = 30)	Group B (n = 30)	p-Value
	Mean ± SD	Mean ± SD	
Preoperative	2.07 ± 1.11	2.50 ± 0.72	0.0803
Postoperative	3.23 ± 0.87	2.54 ± 0.58	0.0006
6 h postoperative	2.64 ± 0.78	2.15 ± 0.55	0.0067

Serum osmolarity was comparable preoperatively and immediately postoperatively (p > 0.05) but was significantly higher in Group B at 6 hours postoperatively (287.3 ± 2.46 vs 284.53 ± 3.72; p = 0.0012) (Table 7).

**Table 7:** Comparison of serum osmolarity and intraoperative variables between the two groups Data are expressed as mean ± SD. Intergroup comparisons were performed using the unpaired Student’s t-test. Corresponding t-scores and p-values were calculated for all parameters. Significant difference was observed only for serum osmolarity at 6 hours postoperatively (t = 3.53, p = 0.0012). A p-value <0.05 was considered statistically significant.

Parameter	Group R (n = 30)	Group B (n = 30)	p-Value
	Mean ± SD	Mean ± SD	
Serum osmolarity			
Preoperative	285.41 ± 4.86	284.33 ± 6.36	0.46
Postoperative	284.46 ± 3.92	285.83 ± 3.62	0.18
6 h postop	284.53 ± 3.72	287.30 ± 2.46	0.0012
Intraoperative IV fluid (mL)	1832.66 ± 191.67	1818.33 ± 183.1	0.76
Maintenance IV fluid (6 h) (mL)	588.33 ± 87.77	576.66 ± 82.76	0.59
Intraop blood loss	416 ± 55.24	406 ± 46.57	0.45
Intraop urine output	419 ± 81.38	405.8 ± 45.05	0.45

Intraoperative variables

The total intraoperative fluid administered and maintenance fluid given during the first 6 postoperative hours were comparable between the groups (p > 0.05).

There was no significant difference in intraoperative blood loss (416 ± 55.24 mL vs 406 ± 46.57 mL; p = 0.45) or urine output (419 ± 81.38 mL vs 405.8 ± 45.05 mL; p = 0.45) between Group R and Group B (Table 7).

## Discussion

The present study compared the effects of RL and BSS on acid-base status and electrolyte profile in patients undergoing emergency exploratory laparotomy. The findings suggest that the use of BSS is associated with a more favorable acid-base profile and lower lactate levels in the early postoperative period.

Large, randomized trials such as the SALT-ED and SMART studies have demonstrated improved clinical outcomes with balanced crystalloids compared to saline. Self et al. [1] in the SALT-ED trial and Semler et al. [11] in the SMART trial reported better outcomes with balanced solutions, thereby supporting the use of physiologically balanced crystalloids in perioperative and critical care settings.

Baseline demographic characteristics and clinical parameters were comparable between the two groups, ensuring homogeneity and minimizing confounding factors. Intraoperative variables such as fluid administration, blood loss, and urine output were also similar, indicating that the observed differences in biochemical parameters were primarily attributable to the type of fluid administered. Similar findings have been reported in previous studies and meta-analyses comparing balanced crystalloids with unbalanced solutions, as demonstrated by Shaw et al. [12] in patients undergoing major abdominal surgery and Song et al. [13] in their meta-analysis of chloride-restrictive versus chloride-rich fluids.

A key finding of the study was the significantly higher pH observed in the BSS group at 6 hours postoperatively, along with significantly higher serum bicarbonate levels both immediately after surgery and at 6 hours after surgery. These findings suggest improved metabolic status with BSS. In contrast, patients receiving RL demonstrated relatively lower pH and bicarbonate levels, indicating a tendency toward metabolic acidosis.

Serum lactate levels were significantly higher in the RL group both immediately postoperatively and at 6 hours postoperatively. It may be attributable to perioperative surgical stress, transient tissue hypoperfusion, and metabolic response to surgery rather than inadequate fluid replacement alone. In all patients, intraoperative fluid administration was standardized and guided according to the Holliday-Segar formula for maintenance requirements, along with replacement of ongoing fluid and blood losses based on hemodynamic parameters, urine output, and estimated blood loss. Although RL contains lactate as a buffer, its metabolism may be impaired in critically ill or hypoperfused patients, potentially contributing to elevated lactate levels. The lower lactate levels observed in the BSS group further support its role in maintaining better metabolic homeostasis, as also emphasized by Raghunathan et al. [14], who highlighted the importance of fluid composition and its metabolic effects in critically ill patients.

Electrolyte analysis revealed that serum sodium and potassium levels were significantly higher in the BSS group at 6 hours postoperatively, although the differences were within clinically acceptable limits. Serum chloride levels remained comparable between the groups, which may be attributed to the relatively short duration of observation and controlled fluid administration. Serum calcium and osmolarity were also higher in the BSS group at 6 hours postoperatively, suggesting a more stable biochemical profile. Balanced solutions are designed to more closely resemble plasma composition and reduce the risk of dilutional acidosis and electrolyte disturbances, as described by Semler and Kellum [15].

From a clinical perspective, the use of BSS in emergency laparotomy patients may help in better preservation of acid-base status and reduction in lactate accumulation, which are important determinants of patient outcomes. However, despite these biochemical advantages, no significant differences were observed in intraoperative hemodynamic parameters or immediate clinical outcomes in this study, a finding consistent with previous randomized trials, as demonstrated by Zampieri et al. [16] in the BaSICS trial.

The findings of the present study suggest that balanced crystalloids are superior to conventional solutions in maintaining acid-base equilibrium. Balanced solutions are designed to more closely resemble plasma composition and reduce the risk of dilutional acidosis and electrolyte disturbances. In contrast, RL, although widely used, may contribute to metabolic derangements under certain clinical conditions.

Limitations

The present study has several limitations. It was conducted at a single center with a relatively small sample size, which may limit the generalizability of the findings. The duration of follow-up was restricted to the early postoperative period (6 hours), and long-term clinical outcomes such as morbidity and mortality were not evaluated. Additionally, variations in the severity of underlying pathology and perioperative physiological status may have influenced metabolic parameters. Further multicentric studies with larger sample sizes and extended follow-up are warranted to validate these findings.

## Conclusions

The present study demonstrates that the use of BSS in patients undergoing emergency laparotomy is associated with modest improvements in acid-base balance and electrolyte profile compared to RL during the early postoperative period. Patients receiving balanced crystalloids exhibited relatively higher pH and bicarbonate levels along with lower lactate concentrations, suggesting better preservation of metabolic parameters.

However, no significant differences were observed in intraoperative hemodynamic variables or immediate clinical outcomes between the two groups. Therefore, while BSSs may offer certain biochemical advantages, the clinical significance of these findings remains uncertain. Larger multicentric randomized trials with longer follow-up and assessment of patient-centered outcomes are required to determine whether these biochemical differences translate into meaningful clinical benefits.
